# Computational Modeling of Interventions and Protective Thresholds to Prevent Disease Transmission in Deploying Populations

**DOI:** 10.1155/2014/785752

**Published:** 2014-06-09

**Authors:** Colleen Burgess, Angela Peace, Rebecca Everett, Buena Allegri, Patrick Garman

**Affiliations:** ^1^MathEcology, Phoenix, AZ 85086, USA; ^2^piTree Software, Metuchen, NJ 08840, USA; ^3^Military Vaccine Agency (MILVAX), Defense Health Headquarters, Falls Church, VA 22042, USA

## Abstract

Military personnel are deployed abroad for missions ranging from humanitarian relief efforts to combat actions; delay or interruption in these activities due to disease transmission can cause operational disruptions, significant economic loss, and stressed or exceeded military medical resources. Deployed troops function in environments favorable to the rapid and efficient transmission of many viruses particularly when levels of protection are suboptimal. When immunity among deployed military populations is low, the risk of vaccine-preventable disease outbreaks increases, impacting troop readiness and achievement of mission objectives. However, targeted vaccination and the optimization of preexisting immunity among deployed populations can decrease the threat of outbreaks among deployed troops. Here we describe methods for the computational modeling of disease transmission to explore how preexisting immunity compares with vaccination at the time of deployment as a means of preventing outbreaks and protecting troops and mission objectives during extended military deployment actions. These methods are illustrated with five modeling case studies for separate diseases common in many parts of the world, to show different approaches required in varying epidemiological settings.

## 1. Introduction

Military personnel are deployed abroad for missions ranging from humanitarian relief efforts to combat actions. While deployed, troops have very specific goals and objectives to achieve within the time frame of their individual deployment rotations, and delay or interruption in these activities due to reasons such as illness and disease transmission can cause operational disruptions, significant economic loss, and stressed or exceeded military medical resources.

Deployed troops function in environments favorable to the rapid and efficient transmission of many viruses—resulting from infection by disease-carrying forces and local populations, high-risk behavior, and inadequate medical care [1]—particularly when levels of protection are suboptimal. It has been shown that, historically, deployed forces have experienced higher rates of hospital admission from infectious diseases than from battlefield combat and noncombat injuries [1] combined.

When immunity among deployed military populations is low, the risk of vaccine-preventable disease outbreaks increases. This increased risk in turn has an impact on troop readiness and achievement of mission objectives. However, the threat of outbreaks can decrease through public health interventions, particularly targeted vaccination, and the optimization of preexisting immunity among the deployed population. High seroprevalence to vaccine-preventable disease and the corresponding protective threshold have the possibility of providing protection against outbreaks to deployed troops.

Here we explore, through the use of computational models of disease transmission, how preexisting immunity compares with vaccination at the time of deployment as a means of preventing outbreaks and protecting troops and mission objectives during extended military deployment actions.

## 2. Methods: Computational Models

On average, deployed military forces consist mainly of healthy, physically-fit individuals ranging in age from 18 to 45 years. Soldiers enter and leave the deployed population via inward and outbound rotation rates and casualty rates, which may vary throughout the duration of the military action. Within military populations there is a distinct structure in which social mixing can be largely within-unit, with a lower level of between-unit contacts occurring in common areas. The deployment tasks of the individual units can vary dramatically, as can their level of contact with local populations. In regions where viruses are circulating at endemic levels, this local contact can significantly affect transmission rates to troops.

Depending on the mission at hand, inward-rotating soldiers are often vaccinated against common vaccine-preventable diseases. Thus these same soldiers may already have significant preexisting immunity to certain viruses when the time comes for subsequent deployments—as a result of prior vaccination during military service, childhood vaccination, or even previous infection and recovery. While epidemiological details may vary based on the virus, this preexisting immunity is generally highly protective—though in some cases protection may wane over years or decades. The presence of preexisting immunity can be determined by blood tests, and evidence of positive serology is assumed to be protective against transmission.

In contrast, vaccine-derived immunity can be highly variable. Issues such as cold-chain failure can severely impact vaccine efficacy, even to the point of complete lack of protection. Under reasonable conditions, however, many vaccines (such as measles and rubella) can confer very high immunity—nearing 95%, 96%, and even 99% efficacy—though this immunity may not be as thorough as that conferred by disease recovery or implied by the existence of positive serology for the virus as evidenced by blood tests. Some vaccines require multiple doses for full protection, and lengthy lags between immunization and protection can be problematic. Vaccination in the presence of existing immunity can be both unnecessary and costly. In addition, for some vaccines, multiple dosing can result in adverse effects; however, data on current preexisting immunity is not always available to medical personnel administering vaccination in a time-frame compatible with deployment.

If the potential protective benefit of relying on preexisting immunity to protect deploying troops is significant enough, it may be feasible to reduce or eliminate mandatory predeployment vaccination—thus also reducing the potential cost and risks associated with adverse events resulting from overvaccination. But how to evaluate the comparative impacts of the two types of protection?

**Figure 1:**
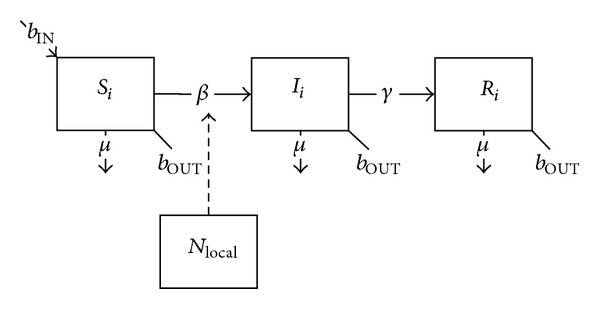
Schematic diagram of the basic SIR model with inbound and outbound rotation, casualty, and contact with local populations.

From a mathematical perspective, simulating preexisting immunity and deployment vaccination requires separate computational approaches. For illustrative purposes, we start with a basic susceptible-infective-removed (SIR) epidemiological model for the transmission of a generic direct-transmissible disease and explore the impacts on this model of the various types of immunity. For this hypothetical situation, soldiers enter unit *i* of the deployed population via inbound rotation (*b*_IN_) and exit through outbound rotation (*b*_OUT_) or casualty (*μ*); upon deployment individuals enter the susceptible (*S*) class and proceed to the infected (*I*) class based on the disease transmission rate (*β*) which is a function of contacts with other infected soldiers within the same (*i*) or different (*j*) units, or with infected individuals within the local population (inc_local_). Infected soldiers enter the removed (*R*) class via recovery (*γ*) and remain there for the duration of their deployment (Figure 1):

(1) dSidt=bINNi−Si(βiiIiNi+∑j≠iβijIjNj+βiLOCALincLOCAL)−μSi−bOUTSi,dIidt=Si(βiiIiNi+∑j≠iβijIjNj+βiLOCALincLOCAL)−γIi−μIi−bOUTIi,dRidt=γIi−μRi−bOUTRi.

**Figure 2:**
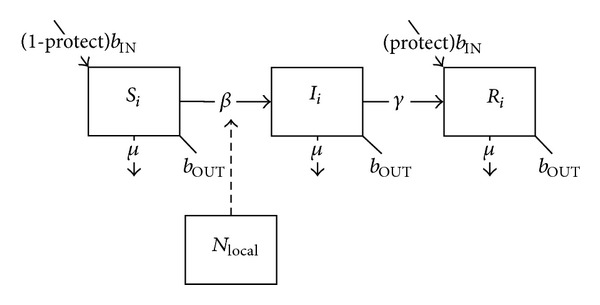
Schematic diagram of the basic SIR model with preexisting immunity.

Since the deployment rotation for individual soldiers is assumed to last between 6 months and one year (independent of the duration of the overall deployment action), we make the simplifying assumption that waning of any preexisting immunity is negligible over such a brief period. We also assume that evidence of positive serology implies complete protection against infection, and thus we can assign the proportion (protect) of inward rotating troops with preexisting immunity “protected” status—that is, these individuals are now in the removed class—and separate them from the segment of the deployed population that may participate in disease transmission. They then retain this protected status for the duration of their individual deployment rotations. The remaining (unprotected) deployed population is thus decreased accordingly so that the risk of transmission among deployed troops overall is also reduced (Figure 2):

(2) dSidt=(1−protect)bINNi−Si(βiiIiNi+∑j≠iβijIjNj+βiLOCALincLOCAL)−μSi−bOUTSi,dIidt=Si(βiiIiNi+∑j≠iβijIjNj+βiLOCALincLOCAL)−γIi−μIi−bOUTIi,dRidt=(protect)bINNi+γIi−μRi−bOUTRi.

**Figure 3:**
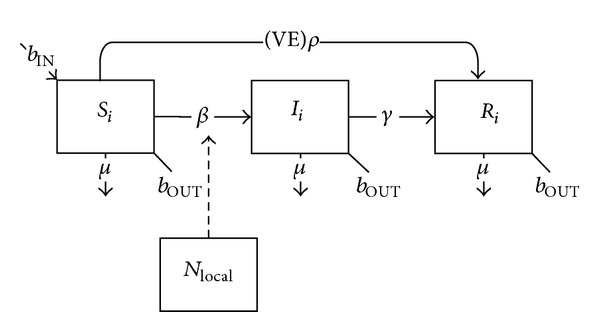
Schematic diagram of the basic SIR model with vaccination at the start of deployment conferring immediate immunity.

For simplicity's sake, we assume that deployment-associated vaccination occurs at day zero of deployment. For vaccines which confer immediate immunity, vaccinated individuals move directly from the susceptible class to the removed class, based on the vaccination coverage rate (*ρ*) (which may be less than 100%) and the vaccine efficacy (VE) (which may also be less than 100%) (Figure 3):

(3) dSidt=bINNi−Si(βiiIiNi+∑j≠iβijIjNj+βiLOCALincLOCAL)−(VE)ρSi−μSi−bOUTSi,dIidt=Si(βiiIiNi+∑j≠iβijIjNj+βiLOCALincLOCAL)−γIi−μIi−bOUTIi,dRidt=(VE)ρSi+γIi−μRi−bOUTRi.

**Figure 4:**
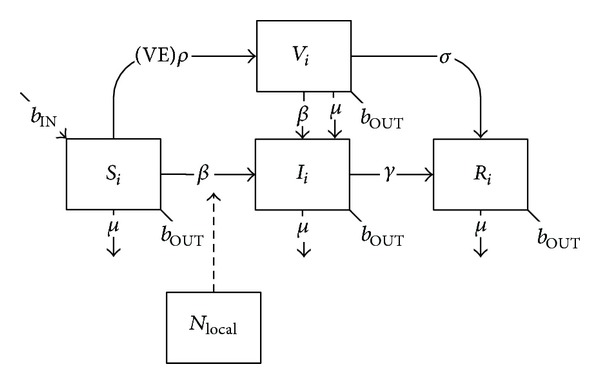
Schematic diagram of the basic SIR model with a time lag between vaccination and protection.

For vaccines with a lag prior to protection (such as hepatitis A) or requiring multiple doses at specific time intervals (such as hepatitis B), one or more additional holding classes (*V*) are required between susceptible and removed classes with rates reflecting vaccine coverage (*ρ*), efficacy (VE), and duration of lag or time between doses (*σ*). In some situations, partially-immunized individuals within these holding classes are protected against disease so long as subsequent doses are administered on time; however for other vaccines immunity is only conferred once all doses are received, and in the meantime individuals in the holding classes are still fully or partially susceptible to transmission (Figure 4):

(4) dSidt=bINNi−Si(βiiIiNi+∑j≠iβijIjNj+βiLOCALincLOCAL)−(VE)ρSi−μSi−bOUTSi,dVidt=(VE)ρSi−Vi(βiiIiNi+∑j≠iβijIjNj+βiLOCALincLOCAL)−σVi−μVi−bOUTVi,dIidt=(Si+Vi)(βiiIiNi+∑j≠iβijIjNj+βiLOCALincLOCAL)−γIi−μIi−bOUTIi,dRidt=σV+γIi−μRi−bOUTRi.

**Figure 5:**
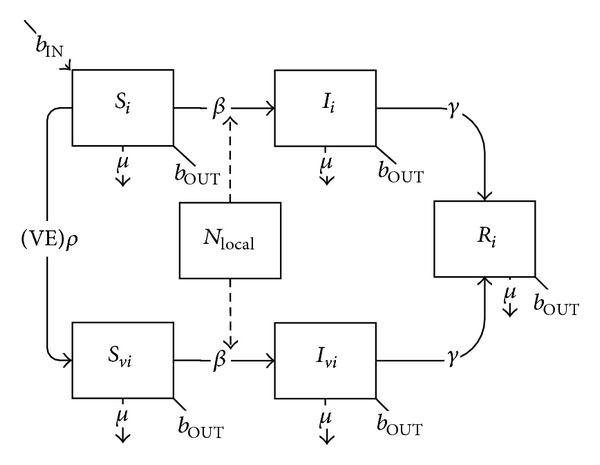
Schematic diagram of the basic SIR model with vaccination with a leaky vaccine.

Finally, for “leaky” vaccines—that is, those for which vaccinated individuals may still become infected, albeit at lower rates of susceptibility (*δ*_*S*_) and with lower resulting infectiousness (*δ*_*I*_)—(such as varicella), an entire separate level of disease classes is required for vaccinated susceptible (*S*_*vi*_) and vaccinated infected (*I*_*vi*_) (Figure 5):

(5) dSidt=bINNi−Si(βii(Ii+(1−δI)Ivi)Ni+∑j≠iβij(Ij+(1−δI)Ivj)Nj+βiLOCALincLOCAL)−(VE)ρSi−μSi−bOUTSi,dIidt=Si(βii(Ii+(1−δI)Ivi)Ni    +∑j≠iβij(Ij+(1−δI)Ivj)Nj    +βiLOCALincLOCAL)   −γIi−μIi−bOUTIi,dSvidt=(VE)ρSi−(1−δs)Svi(βii(Ii+(1−δI)Ivi)Ni+∑j≠iβij(Ij+(1−δI)Ivj)Nj+βiLOCALincLOCAL)−μSvi−bOUTSvi,dIvidt=(1−δs)Svi(βii(Ii+(1−δI)Ivi)Ni+∑j≠iβij(Ij+(1−δI)Ivj)Nj+βiLOCALincLOCAL)−γIvi−μIvi−bOUTIvidRidt=γ(Ii+Ivi)−μRi−bOUTRi.

To evaluate the comparative impacts of preexisting immunity and predeployment vaccination, we look at the* protective threshold*—the specific proportion of the population possessing immunity, above which disease transmission is prevented and below which transmission continues to occur. Specifically, we are looking for the levels of preexisting immunity or vaccination coverage rates which result in a mean annual disease incidence of zero over the duration of the deployment action. To narrow in on this target, we simulate preexisting immunity percentages and predeployment vaccination coverage rates ranging from 0% to 100%. Because epidemiological conditions vary significantly from disease to disease, and from vaccine to vaccine, we illustrate this method by exploring specific case studies of vaccine-preventable viruses that are known to be endemic in many regions of the world, and to which deployed populations may be exposed on a regular basis.

## 3. Results and Discussion: Case Studies

We evaluate all case study disease models illustrated below from the perspective of a deployed military population functioning from a long-standing base, with four types of units having varying levels of contact with local populations ranging from high to negligible daily rates of interaction depending on duty type, based on deployment conditions as described in published literature [2–7].

**Figure 6:**
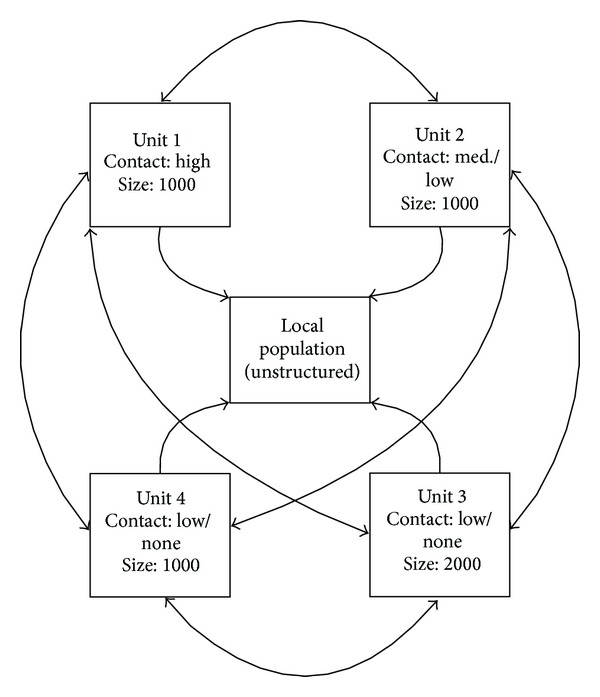
Schematic diagram of the basic deployed population structure and interactions with local populations.

Unit-specific levels of contact with local populations are dependent upon the particular tasks assigned, with some units, such as support troops, spending only the bare minimum of time off-base; in contrast, other units such as Civil Affairs and Special Forces may interact with locals on an almost-daily basis (unpublished data). For this analysis the deployed population is assumed to consist of one unit of 1,000 soldiers with a high daily probability of contact with the local population; one unit of 1,000 soldiers with a medium-to-low daily probability of contact with locals; one unit of 2,000 soldiers with low-to-no daily probability of contact with locals; and one additional unit of 1,000 soldiers, also with low daily probability of contact with the local population (Figure 6).

For long-standing bases, troop housing is assumed to be set up by unit and social mixing is assumed to be “cliquish” (mainly within-unit), though conditions are very crowded and so complete isolation of units is impossible. Mixing within units is assumed to be very homogeneous and high-level, with each individual within the given unit having daily contact of some kind with all other individuals of that same unit.

**Figure 7:**
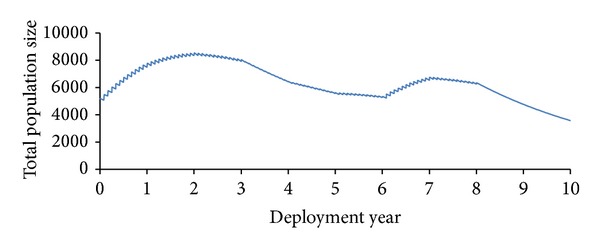
Change in deployed population size over the duration of deployment.

Long-standing bases are assumed to have well-established water purification and food safety systems, with a variety of on-base dining options, though frequently fresh produce is brought in from local areas and nearby countries, increasing the risk of transmission of food-borne illness. We assume the overall deployment action lasts over a period of ten years, and individual soldiers have 12-month deployment rotations. Inbound and outbound rotation rates are assumed to vary over the deployment action [2–7], allowing the overall deployed population to expand and contract in size (Figure 7).

### 3.1. Measles

Measles is a highly infectious viral disease, causing symptoms including fever and cough as well as a blotchy red rash on the face that spreads to become more generalized. Measles virus is transmitted via droplet spread or direct contact with infected individuals and is one of the most highly communicable infectious diseases [8]. The measles incubation period lasts 7 to 18 days from exposure and onset of fever, followed by an infectious period of roughly one week with an attack rate of 3% to 11% in generally healthy populations [9–11]. Measles mortality rates can range from 0.2% to 30%, mainly among young and malnourished children [8]; mortality among healthy adults is negligible.

Live, attenuated measles vaccine is the preferred control measure, a single dose of which results in significant antibody response in 94%–98% of susceptibles [8]. The measles vaccine is now commonly found in the MMR (measles-mumps-rubella) combined vaccine, which is recommended for immunization of all children in the USA at 12–15 months of age, followed by a second dose at school entry [8]. As of 2012, MMR vaccination coverage among children in the USA is estimated at approximately 91% [12].

The transmission of measles shows marked annual seasonality, in addition to periodic large epidemics roughly every two years [13]. Measles infection occurs worldwide, and though the global average measles vaccination coverage was estimated at 84% in 2012 [14], many countries and local regions have coverage rates significantly below this.

Endemic measles transmission was eliminated in the Americas in 2002 [15]; however importation of measles cases from other regions continues to occur. A 1980 study of United States Air Force recruits reported that 22.3% of men and 17.8% of women recruits were susceptible to measles at that time [9], and recent seropositivity data from MILVAX (unpublished) suggest that the level of susceptibility among US Army recruits is currently approximately 8%–16%.

**Figure 8:**
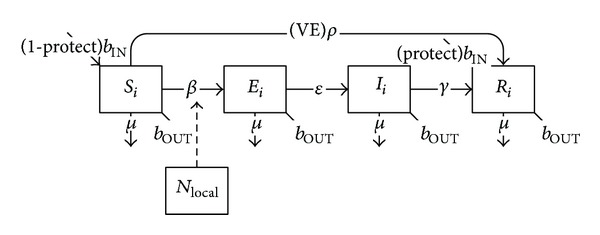
Schematic diagram of measles transmission and vaccination.

The mathematical structure for the respiratory transmission of measles requires the addition of an exposed class to the basic SIR model, to accommodate the incubation stage for the disease. Due to single vaccine dosing and absence of lag prior to protection, vaccinated individuals move directly from the susceptible class to the removed class based upon measles coverage rate and vaccine efficacy (Figure 8, Table 1):

(6) dSidt=(1−protect)bINNi−Si(βiiIiNi+∑j≠iβijIjNj+βiLOCALincLOCAL)−(VE)ρSi−μSi−bOUTSi,dEidt=Si(βiiIiNi+∑j≠iβijIjNj+βiLOCALincLOCAL)−εEi−μEi−bOUTEi,dIidt=εEi−γIi−μIi−bOUTIi,dRidt=(protect)bINNi+(VE)ρSi+γIi−μRi−bOUTRi.

The measles transmission model was based on previously-developed work [17] and was calibrated against historical measles outbreaks for Afghanistan as reported to the World Health Organization [18] under the assumption of full mixing between deployed and local populations.

**Figure 9:**
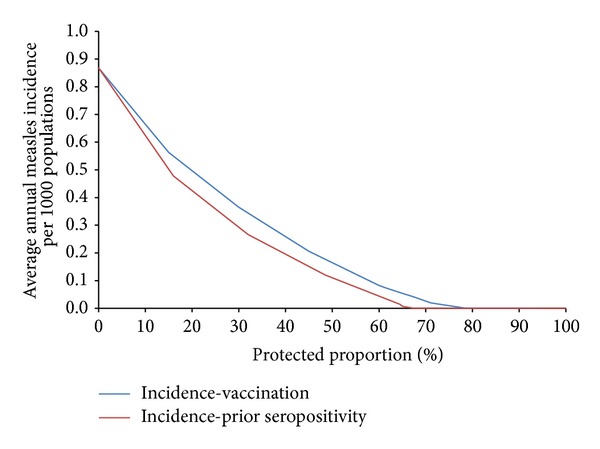
Mean annual measles incidence among deployed populations as a function of protection resulting from preexisting immunity or deployment vaccination.

At low levels of protection, computational simulations of the measles model show that the difference between preexisting immunity and deployment vaccination, in terms of mean annual measles incidence, is minor; however this difference becomes more significant as protection levels increase (Figure 9).

Measles is known to have minimum threshold susceptible population limits, below which transmission will not occur [19]. Model simulations show that this susceptible population limit is attained at 66% preexisting immunity or 79% protection resulting from deployment vaccination. At (and above) these levels of protection, the total number of susceptible soldiers is too low for any individual to acquire infection within the period of deployment—thus defining protective thresholds for immunity. For this particular setting, when preexisting immunity among deploying troops meets or exceeds these thresholds, it is likely that additional deployment vaccination may not be required to protect soldiers from measles transmission.

### 3.2. Rubella

Rubella is a mild febrile viral disease, causing flu-like symptoms as well as a nonraised rash on the face that spreads to the torso and limbs, which closely represents the rash resulting from infection with measles or scarlet fever. As with measles, rubella virus is transmitted via droplet spread or direct contact with infected individuals; however rubella is significantly less infectious [8, 20, 21]. Rubella infection in women in their first trimester of pregnancy can result in congenital rubella syndrome in their unborn infants, potentially causing significant defects or death of the fetus [8] (not included in this case study analysis).

A live, attenuated rubella virus vaccine has been available since 1969, a single dose of which results in significant antibody response in 95%–99% of susceptibles [8, 20, 22]. As with measles, the rubella vaccine is commonly found in the MMR combined vaccine; approximately 90% of children in the United States had received the rubella vaccine as of 2006 [23].

Though rubella transmission shows higher prevalence in winter and spring, it is less affected by seasonality than measles and the magnitude of the seasonal peaks is smaller [13, 24]. Rubella infection occurs worldwide and is considered “universally endemic” in most countries [8]. Though rubella disease is obligatorily reportable to the World Health Organization (WHO), clinical diagnosis of rubella is often inaccurate and is frequently mistaken for measles or scarlet fever—or not diagnosed at all since up to 50% of infections can present without rash [8]. Laboratory confirmation is the only reliable evidence of infection. As a result, rubella incidence as reported to WHO is often significantly lower than actual levels of infection within the community.

After a universal vaccination campaign in 2004, endemic rubella transmission was eliminated in the United States; endemic transmission has also been eliminated in all of the WHO Region of the Americas [14]; however continuing transmission in other regions of the world implies that the US is still at risk of imported cases of rubella, particularly from areas where rubella vaccination programs have not been established [25]. The 1980 study of United States Air Force recruits reported that 18.4% of men and 18.7% of women recruits were susceptible to rubella at that time [9], and recent seropositivity data from MILVAX (unpublished) suggest the level of susceptibility among US Army recruits is currently approximately 15%–22%.

The mathematical structure for the transmission of rubella closely parallels that of measles, as both diseases share common characteristics in terms of their transmission and vaccination (see Figure 8 and (6)), though specific parameter values differ (Table 2).

The structure of the rubella transmission model was based on published models [26, 27] and was qualitatively calibrated against rubella cases reported to the World Health Organization during 1999–2011 for Afghanistan, Iran, Pakistan, Tajikistan, Turkmenistan, and Uzbekistan [28] assuming full mixing between deployed and local populations. Though rubella is a globally reportable disease, it is important to stress that the annual rubella case totals reported to WHO are likely lower than actual infection levels due to underreporting, asymptomatic infections, and misdiagnosis of the disease.

**Figure 10:**
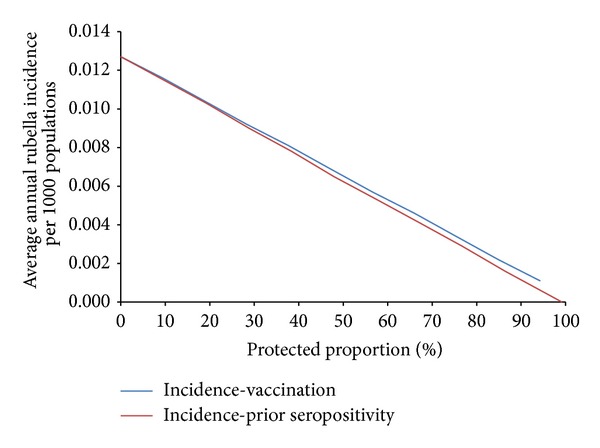
Mean annual rubella incidence among deployed populations as a function of protection resulting from preexisting immunity or deployment vaccination.

Similar to measles, the rubella vaccine has very high efficacy (95%) with full protection conferred immediately after immunization. However, unlike measles, there is no minimum susceptible population limit for rubella. As a result, we can observe rubella transmission persisting all the way to 100% protection (Figure 10). At low levels of protection, computational simulations show that the difference between preexisting immunity and deployment vaccination, in terms of mean annual measles incidence, is negligible; this difference remains minor as protection levels increase, and we find that no distinct protective thresholds exist to bring rubella incidence levels to zero within deployed populations for either type of immunity.

Overall, the simulated mean annual rubella incidence is low—even for scenarios with no immunity—however rubella cases do still occur, up to approximately 150 in total during the 10-year deployment action for no protection whatsoever (results not shown). In light of the endemic nature of rubella infection in countries throughout the world, and in combination with the suboptimal level of preexisting protection against RUB among American Warfighters, it is likely that continuing high levels of vaccination coverage in the absence of elevated preexisting seropositivity is advisable, particularly in combination with efforts to reduce effective contact rates with potentially infected local populations. Even in this case, however, symptomatic and asymptomatic rubella cases will continue to occur among deployed populations, albeit at very low levels.

### 3.3. Varicella

Varicella is a highly contagious disease spread by direct person-to-person contact and airborne droplet transmission, characterized by mild fever and a scabbing rash, though mild or asymptomatic infections may occur. The varicella incubation period lasts two weeks [8, 34], and the infectious period spans 5–10 days [4, 34, 35], with negligible mortality, though fever and constitutional manifestations may be severe in adults. Latent manifestation of the varicella virus can occur in the form of herpes zoster (shingles) and can result in severe pain and neuralgia (zoster is not included in this case study analysis).

A live attenuated varicella vaccine has been licensed for use in the US since 1995, and routine childhood vaccination is recommended at one year of age with a booster at school entry [8, 36]. Vaccine efficacy is estimated at 90% and immediate subsequent immunity is conferred; however breakthrough infections can occur at a reduced rate, with milder symptoms and lower infectivity.

Varicella transmission occurs worldwide, with 95% of individuals in temperate climates experiencing infection by young adulthood [8]. Recent seropositivity data from MILVAX (unpublished) suggest that the level of existing susceptibility among US Army recruits is currently approximately 3%–8%.

**Figure 11:**
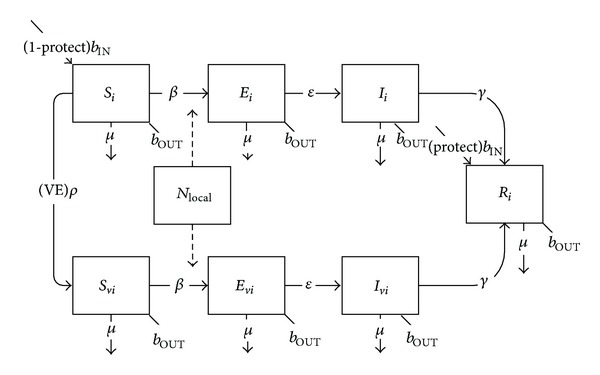
Schematic diagram of varicella transmission and vaccination.

The mathematical structure for the transmission and vaccination of varicella requires the addition of an exposed compartment, along with an extra level of disease classes, to accommodate the leaky nature of varicella vaccine (Figure 11, Table 3):

(7) dSidt=(1−protect)bINNi−Si(βii(Ii+(1−δI)Ivi)Ni+∑j≠iβij(Ij+(1−δI)Ivj)Nj  + βiLOCALincLOCAL)−(VE)ρSi−μSi−bOUTSi,dEidt=Si(βii(Ii+(1−δI)Ivi)Ni +∑j≠iβij(Ij+(1−δI)Ivj)Nj+ βiLOCALincLOCAL)− εEi−μEi−bOUTEi,   dIidt=εEi−γIi−μIi−bOUTIi,dSvidt=(VE)ρSi−(1−δs)Svi(βii(Ii+(1−δI)Ivi)Ni+∑j≠iβij(Ij+(1−δI)Ivj)Nj+ βiLOCALincLOCAL)−μSvi−bOUTSvi,dEvidt=(1−δs)Svi(βii(Ii+(1−δI)Ivi)Ni+∑j≠iβij(Ij+(1−δI)Ivj)Nj+βiLOCALincLOCAL)−εEvi−μEvi−bOUTEvi,dIvidt=εEvi−γIvi−μIvi−bOUTIvi,dRidt=(protect)bINNi+γ(Ii+Ivi)−μRi−bOUTRi.

The structure of the varicella transmission model was based on published models [37] and was calibrated against published historical varicella outbreaks for Romania, Italy, and Pakistan [38–40] assuming full mixing between deployed and local populations.

**Figure 12:**
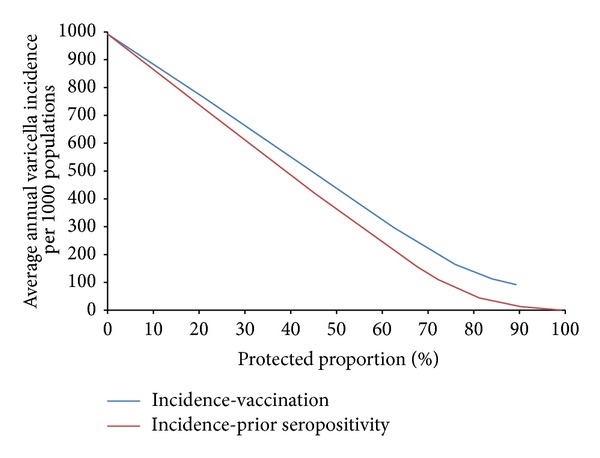
Mean annual varicella incidence among deployed populations as a function of protection resulting from preexisting immunity or deployment vaccination.

At lower levels of protection, computational simulations again show that the difference between preexisting immunity and deployment vaccination, in terms of mean annual varicella incidence, is minor; however, as with measles, this difference becomes more significant as protection levels increase (Figure 12). Protection associated with preexisting varicella seropositivity is assumed to be immediate, life-long, and complete, whereas vaccine-derived immunity is both incomplete and leaky, with a vaccine efficacy of only 90% and probability of breakthrough infection, even in fully-immunized individuals; hence the former can be seen to have a greater impact on incidence particularly as immunity levels increase.

Even as protection levels approach 100%, varicella cases continue to occur and we can see that there are no distinct protective thresholds for the cessation of its transmission within deployed populations. In light of the aggressive varicella attack rate among susceptible individuals, it is likely that continuing high levels of vaccination coverage in the absence of elevated preexisting seropositivity is advisable, perhaps in combination with efforts to reduce effective contact rates with potentially infected local populations.

### 3.4. Hepatitis A

Unlike measles, rubella, and varicella, transmission of hepatitis A is assumed to be dual-mode: via direct- or close-contact between susceptible and infected individuals, and via indirect environmental exposure through the consumption of contaminated food and/or water. Hepatitis A has an incubation period lasting from two to seven weeks [8, 41, 42], and an infectious period lasting approximately three weeks [22, 43]. Once infected, 85% [44] of cases are assumed to be symptomatic, while the remaining 15% are asymptomatic and experience lower levels of infectivity. Hepatitis-associated mortality is assumed to be extremely low [8, 45, 46].

Inactivated hepatitis A vaccine has an efficacy of 99% [42, 43, 45, 47]; however there is a 30-day lag period before full immunity is conferred, during which these newly-vaccinated individuals may become infected. In the US, hepatitis A vaccine is recommended for 1-year-old children[48].

Hepatitis A transmission is generally low in developed countries with good sanitation and hygienic conditions; however transmission can be very high in developing countries with poor conditions, and the majority of children in these regions have been infected with hepatitis A before the age of 10, mostly asymptomatically [49]. In the United States it is estimated that 33% of the population has evidence of prior infection [8], and recent seropositivity data from MILVAX (unpublished) suggest that the level of susceptibility among US Army recruits is currently approximately 66%–73%.

**Figure 13:**
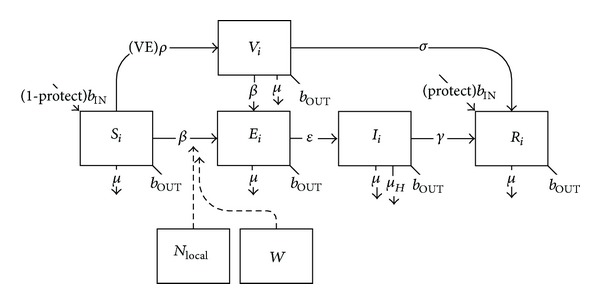
Schematic diagram of hepatitis A transmission and vaccination.

The mathematical structure for the transmission and vaccination of hepatitis A requires the inclusion of a separate input corresponding to the environmental reservoir for hepatitis A, along with an additional vaccinated class, to accommodate the lag period between vaccination and full protection and the potential for continued transmission during this period (Figure 13, Table 4):

(8) dSidt=(1−protect)bINNi−Si×(βiiIiNi+∑j≠iβijIjNj+βiLOCALincLOCAL+βiWW)−(VE)ρSi−μSi−bOUTSi,dVidt=(VE)ρSi−Vi×(βiiIiNi+∑j≠iβijIjNj+βiLOCALincLOCAL+βiWW)−σVi−μVi−bOUTVi,dEidt=(Si+Vi)×(βiiIiNi+∑j≠iβijIjNj+βiLOCALincLOCAL+βiWW)−εEi−μEi−bOUTEi,dIidt=εEi−γIi−μIi−μHIi−bOUTIi,dRidt=(protect)bINNi+σV+γIi−μRi−bOUTRi.

The structure of the hepatitis A transmission model was based on published models [41, 50] and was calibrated against historical hepatitis A outbreaks for Saudi Arabia, Qatar, and Palestine as reported in published literature [51–53] assuming full mixing between deployed and local populations.

**Figure 14:**
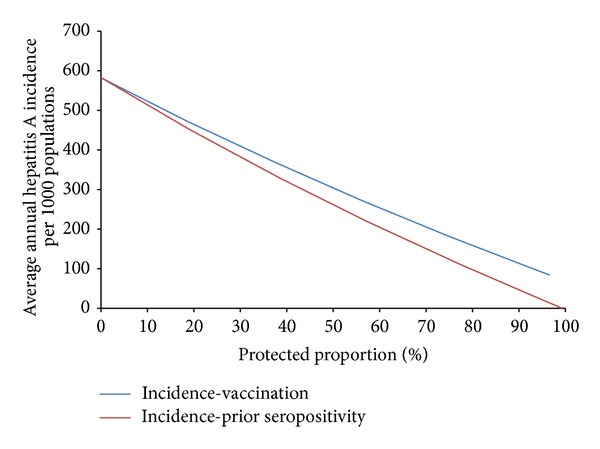
Mean annual hepatitis A incidence among deployed populations as a function of protection resulting from preexisting immunity or deployment vaccination.

As in the previous case studies, computational simulations again show that the difference between preexisting immunity and deployment vaccination, in terms of mean annual hepatitis A incidence, becomes more significant as protection levels increase, with preexisting protection having a greater impact than deployment vaccination (Figure 14).

In spite of high vaccine efficacy (99%), there is a 30-day lag after vaccination before full protection is imparted on immunized soldiers—and within this lag period transmission of hepatitis A resulting from effective contact with infected individuals or contaminated food is still possible. This long delay dramatically reduces the effectiveness of vaccination at the beginning of deployment.

For hepatitis A, again model simulations indicate that there is no protective threshold which prevents disease transmission. Even 100% vaccination coverage prevents only 85%–90% of simulated hepatitis A incidence (results not shown), due to the lengthy time-lag between vaccination and full protection. Sensitivity analyses (results not shown) indicate that exposure to contaminated food sources is a higher risk factor for hepatitis-A transmission than contact with infected individuals; thus any efforts to reduce this exposure could significantly decrease disease incidence among deployed troops. Also, although vaccination provides less protection against hepatitis A transmission among deployed populations than does preexisting seropositivity, it is likely that immunizing troops with hepatitis A vaccine at least 30 days prior to deployment may improve this protection by eliminating (or drastically reducing) the possibility of exposure to contamination during the efficacy lag period.

### 3.5. Hepatitis B

Hepatitis B is the most serious type of viral hepatitis, causing potentially life-threatening liver infection, chronic liver disease, and risk of death due to cirrhosis of the liver and liver cancer. Hepatitis B is transmitted by direct contact with blood or other bodily fluids of infected individuals, similar to human immunodeficiency virus (HIV), though with dramatically greater infectivity. Unlike HIV, the hepatitis B virus can survive outside the body for at least 7 days. During this time, the virus can still cause infection if it enters the body of a person who is not protected by the vaccine or immunity resulting from recovery from prior infection. Hepatitis B is not spread by contaminated food or water and cannot be spread by casual contact.

50%–90% of patients with acute hepatitis B infection experience subclinical illness that may remain undetected; the remaining acute patients develop symptoms that can include fever, fatigue, anorexia, nausea, body aches, jaundice, and (rarely) acute liver failure [8], which can progress to chronic infection at a rate dependent upon the age at infection. Most individuals who are infected as adults will develop acute hepatitis and be able to clear the disease; approximately 1–10% of individuals newly-infected as adults will develop chronic hepatitis [55, 56].

A vaccine against hepatitis B has been available since 1982, which is 95-96% effective in preventing infection while conferring long-term (possibly lifelong) immunity [8, 57–59]. The vaccination series for newborns consists of 3 doses: birth, 1-2 months of age, and 6–18 months of age; the dosing schedule for unvaccinated adults also includes 3 doses, with the second given 4 weeks after the first and the third 3–5 months after the second [57]. As of 2001, childhood vaccination coverage in the US with all three doses averaged 88%-89% [60], and 2010 coverage among adults was estimated at 42% [61]. Recent seropositivity data from MILVAX (unpublished) suggest that the level of susceptibility among US Army recruits is currently approximately 41–55%.

Prevalence and incidence rates of hepatitis B vary with location as well as cultural behaviors, though seasonal patterns of transmission are absent [62]. In Western Europe and North America it is estimated that less than 1% of the population is chronically infected with hepatitis B [8, 56]. In the United States prevalence is significantly higher for those with military background [61] due to risk factors such as communal living conditions and exposure in developing countries where viral hepatitis is endemic [63]. In combat deployments, additional risk results from the use of fresh whole blood transfusion when demands for massive transfusions outstrip existing supplies [64]. This is of particular relevance when immunization against hepatitis B is less than optimal and exposure to the virus has already occurred during the deployment, as the incubation period can be on the order of months and the majority of infections are subclinical [8].

**Figure 15:**
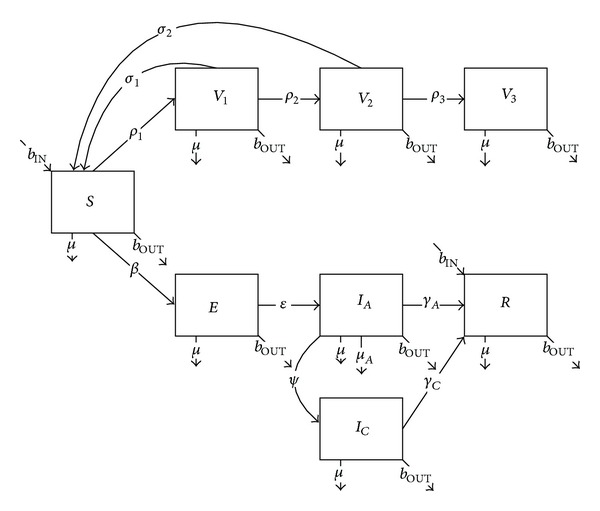
Schematic diagram of hepatitis B transmission and vaccination.

The mathematical structure for the transmission of hepatitis B requires that the infected class be split into acute and chronic compartments to accommodate the separate epidemiological characteristics. The model also requires the addition of three distinct holding compartments representing the separate doses required for immunity, starting with dose 1 at the time of deployment (Figure 15, (9), Table 5). In the absence of clinical data along these lines, we assume within the model that partially immunized individuals are protected at the full vaccine efficacy so long as subsequent doses are administered on schedule:

(9) dSidt=bIN(1−protect)Ni−Si(βii(IAi+ICi)Ni+∑j≠iβij(IAj+ICj)Nj   + βiLOCALincLOCAL)−ρ1iSi+σ1(1−ρ2i)V1i+σ2(1−ρ3i)V2i−μSi−bOUTSi,dV1idt=ρ1iSi−σ1V1i−μV1i−bOUTV1i,dV2idt=σ1ρ2iV1i−σ2V2i−μV2i−bOUTV2i,dV3idt=σ2ρ3iV2i−μV3i−bOUTV3i,dEidt=Si(βii(IAi+ICi)Ni+∑j≠iβij(IAj+ICj)Nj    + βiLOCALincLOCAL)−εEi−μEi−bOUTEi,dIAidt=εEi−γAIAi−ψIAi−μAIAi−μIAi−bOUTIAi,dICidt=ψIAi−γCICi−μICi−bOUTICi,dRidt=bIN(protect)Ni+γAIAi+γCICi−μRi−bOUTRi,dNidt=bINNi−μAIAi−μNi−bOUTNi.

The structure of the hepatitis B transmission model was based on published models [65]. Local HBV prevalence in the model is set at 5%, based on estimated carrier rates within Middle Eastern countries with moderate vaccination coverage (68–90%) and intermediate endemicity and countries with lower vaccination coverage (under 68%) and higher endemicity [66], as well as global regions with mixed transmission modes and intermediate carrier rates [67].

**Figure 16:**
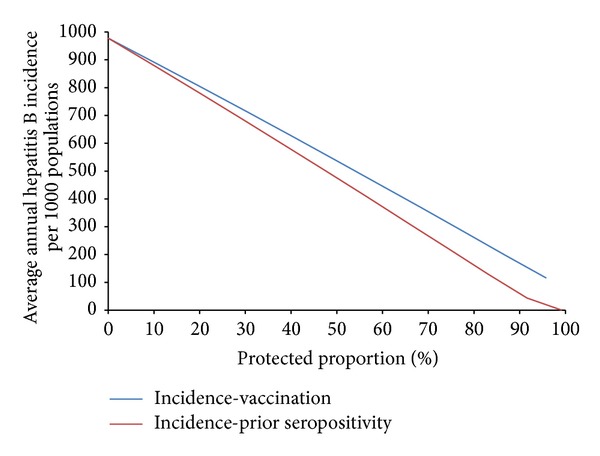
Mean annual hepatitis B incidence among deployed populations as a function of protection resulting from preexisting immunity or deployment vaccination.

As with the previous case studies, computational simulations again show that the difference between preexisting immunity and deployment vaccination, in terms of mean annual hepatitis B incidence, becomes more significant as protection levels increase, with preexisting protection having a greater impact than deployment vaccination (Figure 16).

For hepatitis B, simulations show that no distinct protective thresholds exist for the cessation of transmission within deployed populations for either type of immunity. Though three doses of vaccine are required for lasting immunity, partially immunized individuals are assumed to be protected at the full vaccine efficacy so long as subsequent doses are administered on schedule; thus the difference between the two types of immunity is most likely a result of the 4% of vaccinated individuals failing to receive protection due to imperfect vaccine efficacy (we assume that soldiers return to the base medical facility on time to receive all doses).

Protection associated with preexisting hepatitis B seropositivity resulting from previous vaccination or recovery from infection is assumed to be immediate, life-long, and complete, whereas vaccine-derived immunity is both incomplete and lengthy to acquire, with a vaccine efficacy of only 96% and a total of six months to complete the entire three-dose vaccine course. In light of the persistent level of chronic carriers worldwide, and in combination with the only moderate level of preexisting protection against hepatitis B currently among American Warfighters, it is likely that continuing high levels of vaccination coverage in the absence of elevated preexisting seropositivity is advisable, particularly in combination with efforts to reduce effective contact rates with potentially infected local populations. Even in this case, however, hepatitis B cases could continue to occur among deployed populations, particularly if partially immunized individuals do not experience full protection between doses.

## 4. Conclusions: Limitations and Next Steps

All models are abstractions of reality and as such involve necessary simplifications in order to produce a numerical model which can be simulated. Deterministic models, in particular—as in the case studies above—can obscure transmission dynamics at extremely low incidence levels implying that protective thresholds may possibly occur at lower levels of preexisting immunity or deployment vaccination. Also the case study population structure assumes homogeneity within subgroups, which can be inappropriate for populations structured with a high degree of segregation between groups, particularly in terms of implementing highly focused outbreak responses. However, depending on deployed conditions, this may not be an issue for the case study populations as within-unit mixing is assumed to occur within relatively confined areas and may closely approximate homogeneity. Finally, parameter values implemented during analyses were derived from a variety of data sources, some of which may not be representative of populations directly compatible with deployed US military populations. Sensitivity and uncertainty analyses performed during the case studies (results not shown) have explored these issues for the specific diseases and settings described above.

There has also been some indication that detection of preexisting seropositivity to a virus may not be equivalent to full protection against transmission (see, e.g., [73]). Reduction or lack of protection in individuals with prior serology for a virus could dramatically affect model predictions in terms of the relative benefits of deployment vaccination.

However the exploration of the protective threshold, even in the presence of model limitations, can still provide valuable insight into the best way in which to protect deploying troops from transmission of vaccine-preventable disease. Building upon these results, we can now incorporate additional aspects unique to this setting—such as the risk of adverse events resulting from the administration of multiple vaccine doses, delay in implementation of full vaccine course, or reduced vaccine efficacy due to harsh field conditions—along with cost calculations to further expand the comparison between preexisting immunity and vaccination at the time of deployment as a means of preventing outbreaks and protecting troops and mission objectives during extended military deployment actions.

## Figures and Tables

**Table 1:** Model parameters for measles transmission model.

Parameter	Description	Value	Source
*b*_IN_	Inward troop rotation rate	Varies over duration of deployment	[3, 16]
*b*_OUT_	Outward troop rotation rate	Varies over duration of deployment	[3, 16]
*β*_*ii*_	Effective transmission rate within unit *i*	Function of attack rate	
*β*_*ij*_	Effective transmission rate between units *i* and *j*	Function of attack and contact rates	
*β*_*i*LOCAL_	Effective transmission rate between unit *i* and local population	Function of attack rate, contact rate, and local measles incidence	
*χ*	Measles attack rate (soldiers)	7% (3%–11%)	[17]
*c*_*i*_	Contact rate with local population for unit *i*	Varies by unit	
*C*_*ij*_	Contact rate between unit *i* and unit *j*	Varies by unit	
inc_LOCAL_	Measles incidence rate for local population	Varies over duration of deployment	[18]
*ρ*_*i*_	Vaccination rate for unit *i* (95% vaccine efficacy)	Scenario-dependent	
*μ*	Background casualty rate for all units	0.0007825 1/day	[16]
1/*ε*	Duration of measles incubation period	9 days	[17]
1/*γ*	Duration of measles infectious period	7 days	[17]

**Table 2:** Model parameters for rubella transmission model.

Parameter	Description	Value	Source
*b*_IN_	Inward troop rotation rate		[3, 16]
*b*_OUT_	Outward troop rotation rate		[3, 16]
protect	Proportion of population protected via prior seropositivity	Scenario-dependent	
*β*_*ii*_	Effective transmission rate within unit *i*	Function of attack rate	
*β*_*ij*_	Effective transmission rate between units *i* and *j*	Function of attack and contact rates	
*β*_*i*LOCAL_	Effective transmission rate between unit *i* and local population	Function of attack rate, contact rate, and local RUB incidence	
*c*_*i*_	Contact rate with local population for unit *i*	Varies by unit	
*C*_*ij*_	Contact rate between unit *i* and unit *j*	Varies by unit	
*χ*	RUB attack rate (for soldiers)	0.019% (0.019%–8%)	[29–32]
inc_LOCAL_	RUB incidence rate for local population	Varies over time	[28, 33]
1/*ε*	Duration of RUB incubation period	12 days (10–21 days)	[21, 26, 27]
1/*γ*	Duration of RUB infection period	12.5 days (11–14 days)	[20, 26, 27]
*μ*	Casualty rate for all units (nondisease related)	0.0007825	[16]
*ρ*	Vaccination rate for dose 1 (95% vaccine efficacy)	Scenario-dependent	

**Table 3:** Model parameters for varicella transmission model.

Parameter	Description	Value	Source
*b*_IN_	Inward troop rotation rate	Varies over duration of deployment	[3, 16]
*b*_OUT_	Outward troop rotation rate	Varies over duration of deployment	[3, 16]
*β*_*ii*_	Effective transmission rate within unit *i*	Function of attack rate	
*β*_*ij*_	Effective transmission rate between units *i* and *j*	Function of attack and contact rates	
*β*_*i*LOCAL_	Effective transmission rate between unit *i* and local population	Function of attack rate, contact rate, and local varicella incidence	
*δ*_*S*_	Proportional reduction in varicella susceptibility after vaccination	97% (50%–100%)	[37]
*δ*_*I*_	Proportional reduction in varicella infectivity after vaccination	50% (20%–100%)	[37]
*χ*	Varicella attack rate (soldiers)	50% (30%–90%)	[8, 38]
*c*_*i*_	Contact rate with local population for unit *i*	Varies by unit	
*C*_*ij*_	Contact rate between unit *i* and unit *j*	Varies by unit	
inc_LOCAL_	Varicella incidence rate for local population	Varies over duration of deployment	[38–40]
*ρ*_*i*_	Vaccination rate for unit *i* (90% vaccine efficacy)	Scenario-dependent	
*μ*	Background casualty rate for all units	0.0007825 1/day	[16]
1/*ε*	Duration of varicella incubation period	14 days	[37]
1/*γ*	Duration of varicella infectious period	7 days	[37]

**Table 4:** Model parameters for hepatitis A transmission model.

Parameter	Description	Value	Source
*b*_IN_	Inward troop rotation rate	Varies over duration of deployment	[3, 16]
*b*_OUT_	Outward troop rotation rate	Varies over duration of deployment	[3, 16]
*β*_*ii*_	Effective transmission rate within unit *i*	Function of attack rate	
*β*_*ij*_	Effective transmission rate between units *i* and *j*	Function of attack and contact rates	
*β*_*i*LOCAL_	Effective transmission rate between unit *i* and local population	Function of attack rate, contact rate, and local measles incidence	
*β*_*iW*_	Effective transmission rate due to environmental exposure	Function of attack rate, consumption of local food, and proportion of local food that is contaminated	
*r*	Proportion of infections that are asymptomatic	85% (76%–97%)	[44]
*θ*	Proportional reduction in infectivity for asymptomatic individuals	10% (0%–25%)	[44]
*χ*	Hepatitis A attack rate (soldiers) via direct transmission	0.82% (0.82%–1.3%)	[44]
*χ*_*W*_	Hepatitis A attack rate (soldiers) via environmental consumption	2.2% (2.2%–3.5%)	[44]
*c*_*i*_	Contact rate with local population for unit *i*	Varies by unit	
*C*_*ij*_	Contact rate between unit *i* and unit *j*	Varies by unit	
*c*_*iW*_	Contact rate with environmental reservoir	Varies by unit	
inc_LOCAL_	Hepatitis A incidence rate for local population	Varies over duration of deployment	[51–53]
*ρ*_*i*_	Vaccination rate for unit *i* (90% vaccine efficacy)	Scenario-dependent	
1/*ψ*	Duration of lag prior to full vaccine-induced immunity	30 days	[44]
*μ*	Background casualty rate for all units	0.0007825 1/day	[16]
*μ*_*H*_	Hepatitis A case fatality rate	0.3% (0.1%–0.8%)	[8, 45, 46]
1/*ε*	Duration of hepatitis A incubation period	14 days	[41, 42]
1/*γ*	Duration of hepatitis A infectious period	21 days	[42, 54]
*W*	Proportion of local food that is contaminated	20% (5%–50%)	Estimated

**Table 5:** Model parameters for hepatitis B transmission model.

Parameter	Description	Value	Source
*b*_IN_	Inward troop rotation rate		[3, 16]
*b*_OUT_	Outward troop rotation rate		[3, 16]
*β*_*ii*_	Effective transmission rate within unit *i*	Function of attack rate	
*β*_*ij*_	Effective transmission rate between units *i* and *j*	Function of attack and contact rates	
*β*_*i*LOCAL_	Effective transmission rate between unit *i* and local population	Function of attack rate, contact rate, and local HBV prevalence	
*c*_*i*_	Contact rate with local population for unit *i*	Varies by unit	
*C*_*ij*_	Contact rate between unit *i* and unit *j*	Varies by unit	
*χ*	HBV attack rate (for soldiers)	25% (11%–71%)	[68–71]
inc_LOCAL_	HBV prevalence rate for local population	5%	[18, 66, 67]
1/*ε*	Duration of HBV incubation period	90 days (60–90 days)	[57]
1/*γ*_*A*_	Duration of HBV acute infection period	28 days (23–28 days)	[72]
1/*γ*_*c*_	Duration of HBV chronic infection period	40 years (lifelong)	Calibrated
*ψ*	Conversion rate from acute to chronic HBV infection	5% (2%–90%)	[65]
*μ*	Casualty rate for all units (nondisease related)	0.0007825	[16]
*μ*_*A*_	Acute infection mortality rate for all units	1% (0.2%–1%)	[57]
*ρ*_1_, *ρ*_2_, *ρ*_3_	Vaccination rate for doses 1, 2, and 3 (96% vaccine efficacy)	Scenario-dependent	
1/*σ*_1_	Duration of *V*_1_ status prior to second dose vaccination	28 days	[57]
1/*σ*_2_	Duration of *V*_2_ status prior to third dose vaccination	150 days	[57]
